# Lipid Oxidation Assessed by Indirect Calorimetry Predicts Metabolic Syndrome and Type 2 Diabetes

**DOI:** 10.3389/fendo.2018.00806

**Published:** 2019-01-10

**Authors:** Arturo Pujia, Elisa Mazza, Yvelise Ferro, Carmine Gazzaruso, Adriana Coppola, Patrizia Doldo, Rosa Daniela Grembiale, Roberta Pujia, Stefano Romeo, Tiziana Montalcini

**Affiliations:** ^1^Department of Medical and Surgical Science, University Magna Grecia, Catanzaro, Italy; ^2^Clinical Institute “Beato Matteo”, Vigevano, Italy; ^3^Department of Health Science, University Magna Grecia, Catanzaro, Italy; ^4^Department of Molecular and Clinical Medicine, University of Gothenburg, Gothenburg, Sweden; ^5^Nutrition Unit, Department of Clinical and Experimental Medicine, University Magna Grecia, Catanzaro, Italy

**Keywords:** lipid oxidation, metabolic syndrome, diabetes, respiratory quotient, calorimetry

## Abstract

**Purpose:** Diabetes has been linked to an impaired ability to oxidize fatty acids. Fat oxidation can be assessed clinically by a respiratory quotient measurement during fasting. We hypothesized that a respiratory quotient might predict metabolic syndrome and type 2 diabetes onset.

**Methods:** In this longitudinal study we used an existing database of 233 individuals who had complete nutritional and biochemical data at baseline and after 12-month follow-up. All participants underwent an indirect calorimetry to measure the respiratory quotient. We excluded participants with diabetes, metabolic syndrome, chronic diseases, and those who had changed food habits in the previous 3 months. Only 88 subjects met the inclusion criteria.

**Results:** Two individuals developed type 2 diabetes and 10 metabolic syndrome after 1 year. Participants in the high respiratory quotient group (>0.91) had a higher incidence of metabolic syndrome/diabetes than those in the low quotient group (25 vs. 8% *p* = 0.04). In this group, mean basal respiratory quotient was 0.97 ± 0.04. In the high respiratory quotient group, Kaplan-Meier curves showed a greater probability of having metabolic syndrome/diabetes than those in the low respiratoryquotient group (log Rank χ^2^-test = 8.44; *p* = 0.004). A multivariable Cox proportional hazards model demonstrated that energy expenditure and weight increase did not predict metabolic syndrome/diabetes [HR (95% CI) = 1 (0.996–1.005), *p* = 0.86 and 3.9 (0.407–38.061), *p* = 0.23, respectively).

**Conclusions:** A greater probability of metabolic syndrome/diabetes was found in individuals with a basal respiratory quotient of >0.91 than in those with a respiratoryquotient of ≤ 0.91 after 1 year. In the short-term anthropometric measurements and their variation overtime were not correlated with metabolic syndrome/diabetes.

## Introduction

Metabolic Syndrome (MetS) and Type 2 diabetes (T2D) are clinical conditions involving the impaired uptake and utilization of glucose, altered lipid metabolism, and the disruption of the metabolic signaling pathways that regulate insulin secretion from pancreas (1). Several reports support the notion that an increase in plasma free fatty acids (FFAs) is key in the development of insulin resistance and T2D (2–4). When the Fatty acid (FA) oxidation capacity in the muscle decreases, the intramyocellular lipid concentration increases (5). It has been found that an increase in diacylglycerol species in insulin resistant obese and T2D subjects stimulates the secretion of insulin by the pancreas to maintain a normal glucose level (6). Under chronic hyperinsulinemia, FA oxidation progressively decreases with a consequent alteration in glucose transport, and/or phosphorylation pathways associated with normal glucose uptake (5).

Taken together, these studies confirm that muscle insulin resistance represents a major factor in the pathogenesis of T2D.

Fat oxidation can be assessed clinically by measuring respiratory quotient (RQ), which is the ratio of the carbon dioxide expired to the oxygen consumed during indirect calorimetry. High RQ values are indicative of low fat oxidation and high carbohydrate oxidation (7). RQ measured during fasting reflects a period of high dependency on endogenous FFAs for fuel. Thus, if an altered ability to oxidize FA represents an important contributor to the genesis of insulin resistance, assessing the capacity to burn fat in human subjects by RQ measurements might be a predictor of MetS and T2DM.

In this study we sought to determine longitudinally whether subjects not affected by MetS and T2D with a high fasting RQ would have a higher risk of developing these clinical conditions than those with a lower fasting RQ and, consequently whether RQ during fasting would predict MetS and T2D.

## Subjects and Method

In this retrospective, longitudinal study, the population consisted of a sample of non-MetS/T2D, white subjects who were undergoing health-screening tests at our outpatient Nutrition Clinic at the “Mater Domini” University Hospital in Catanzaro.

We used the data available for research purposes on existing database. The sample included 233 individuals who had complete nutritional and biochemical data at baseline and after 12-month follow-up, including an indirect calorimetry assessment at baseline.

Individuals were excluded if they had T1D or T2D, MetS or followed a special diet or changed their food habits and/or used any dietary supplements in the 3 months prior to the follow-up visit. All patients included in the study were not suffering from any chronic diseases (like stage 2–5 chronic kidney disease, end stage liver failure, chronic obstructive pulmonary disease, thyroid dysfunction, congestive heart failure, cancer), cardiovascular disease, and were not taking any drug which could affect respiratory gas exchange (like anti-obesity medications, psychotropic drugs, and chronotropic agents), as determined by medical history, a physical examination, and laboratory tests (8).

**Figure 1:**
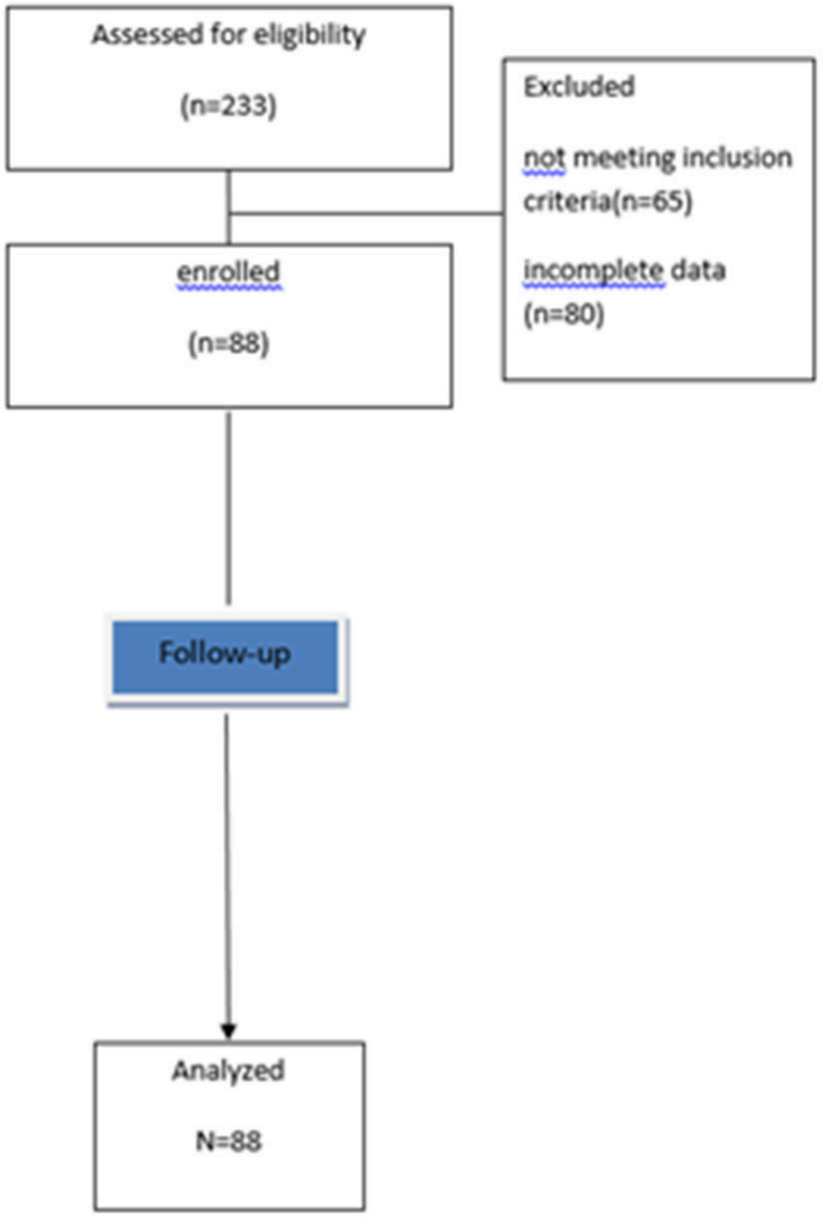
Study flow-chart.

Therefore, in this study we enrolled 88 subjects (study flow-chart, Figure 1).

Written informed consent was obtained from all participants. The investigation conforms to the principles outlined in the Declaration of Helsinki. The protocol was approved by the local ethics committee at the “Mater Domini” University Hospital in Catanzaro, Italy (projects codes 2013-1/CE).

### Outcomes

The purpose of this study was to assess whether RQ, measured at first visit, would predict the MetS/T2DM onset after 12 months. The outcomes of interest were MetS and T2D. MetS was defined according to the guidelines of the National Cholesterol Education Program's Adult Treatment Panel III (NCEP ATP III) (with at least three or more abnormalies) (9).

Diagnosis of T2D was based on use of blood-glucose lowering medication or an elevated fasting blood glucose ≥126 mg/dl (≥7.0 mmol/L).

### Other Covariates

We assessed the presence of the known classical cardiovascular (CV) risk factors. The following criteria were used to define the distinct CV risk factors: hyperlipidemia: total cholesterol >200 mg/dL and/or triglycerides >200 mg/dL or lipid lowering drugs use; hypertension: systolic blood pressure ≥130 mmHg and/or diastolic blood pressure ≥85 mmHg or antihypertensive treatment; obesity: body mass index (BMI) ≥30 kg/m^2^; smoking (current smoker): who has smoked more than 100 cigarettes in their lifetime and smokes cigarettes every day or some days (10, 11).

### Anthropometric Measurements

Body weight was measured before breakfast after a 12 h overnight fast with the subjects lightly dressed, subtracting the weight of clothes. Body weight was measured with a calibrated scale and height measured with a wall-mounted stadiometer. BMI was calculated with the following equation: weight (kg)/height (m)^2^. Participants were classified as weight stable if they maintained a body weight within 5% of baseline weight at 1-year follow-up.

Waist circumferences and hip circumferences (WC and HC) were measured with a non-stretchable tape. Bioelectrical impedance analysis (BIA) (BIA-101, Akern srl, Florence, Italy) was performed to estimate the percentage of Fat Mass (FM) (8, 12).

### Biochemical Evaluation

Venous blood was collected after fasting overnight into vacutainer tubes (Becton & Dickinson, Plymouth, England) and centrifuged within 4 h. Serum glucose, total cholesterol, high density lipoprotein (HDL)-cholesterol, triglycerides, and creatinine were measured with Enzymatic colorimetric test. Low-density lipoprotein (LDL) cholesterol level was calculated by the Friedewald formula. A strict system of quality control (QC) was observed at all levels such as for the collection of the specimen, its processing, instrumentation, and its maintenance. Quality control included testing normal and abnormal controls for each assessment at least daily to monitor the analytical process. Initial calibration and routine maintenance were carried out periodically as recommended by the manufacturers.

#### RQ Assessment—Indirect Calorimetry

RQ and the Resting Energy Expenditure (REE) were measured by Indirect Calorimetry using the open circuit technique (Viasys Healthcare, Hoechberg, Germany). All tests were performed after fasting overnigh, between hours of 7 a.m. and 8:30 a.m. after 48 h abstention from exercise, in a sedentary position. The participant rested quietly for 30 min in an isolated room at a controlled temperature (21–24°C). Respiratory gas exchange was measured within a canopy circuit for at least 30 min, until steady state was achieved. The calorimeter quantifies the volume of O_2_ inspired and CO_2_ expired by the subject. Resting Energy Expenditure is calculated by the Weir formula. RQ was calculated as CO_2_ production/O_2_ consumption. Criteria for a valid measurement was at least 15 min of steady state, with < 10% fluctuation in minute ventilation and oxygen consumption and < 5% fluctuation in RQ (8).

### Statistical Analysis

Data are reported as means ± standard deviations (SD). In this study, to find more than 10% difference in MetS/T2D prevalence between the highest and lowest RQ group, considering a prevalence of MetS of 25% and T2D of 5%, with 80% power on a twosided level of significance, a minimum of 49 subjects for each group were required.

According to the mean RQ value of those participants who developed MetS/T2D, we categorized the enrolled population into the following two groups: High RQ (>0.91) and Low RQ (≤ 0.91).

A chi-square test was performed to analyze the difference in prevalence between participants with and without MetS/T2D and RQ High/Low, and a Fisher Exact test was used in the case of an expected number of frequencies fewer than 5.

Pearson's correlations was used to identify the variables [i.e., age, BMI, WC, WC increase (binary), weight, weight increase >5%—(binary; ΔW > 5%), FM, RQ, RQ categories (binary, High and Low), REE, glucose, total cholesterol, HDL, Triglycerides, creatinine, SBP, and DBP] correlated with MetS/T2D as a binary variable, given that the continuous variables were normally distributed.

An independent samples *t*-test was used to compare the means of these two groups.

The Kaplan-Meier estimation of survival curves with Mantel-Cox log-rank univariate analysis was performed to identify a different probability of having MetS/T2D between High and Low RQ groups. A multivariable Cox proportional hazards model with time to incident MetS/T2D within 1 year, as the outcome of the study, was used to express how the baseline variables, including medications and gender, simultaneously predicted the main outcome. Hazards ratios (HR) and 95% confidence intervals (CI) were determined. Since, at univariate analysis, REE and ΔW > 5% correlated with RQ and MetS/T2D, respectively, these variables were also entered in the model. Furthermore, a Kaplan-Meier estimation of survival curves was performed to identify differences between participants who were or not Weight Stable.

Significant differences were assumed to be present at *p* < 0.05 (two-tailed). All comparisons were performed using SPSS 25.0 for Windows (S. Wacker Drive, Chicago, IL 60606, United States).

## Results

In the whole sample population the mean age was 60 ± 12 years. The mean basal RQ was 0.87 ± 0.08 and 0.86 ± 0.07 in female and male, respectively (*p* = 0.32). A total of 10 individuals developed MetS and two T2D. The mean basal RQ value was 0.91 ± 0.09 in those who developed MetS/T2D.

Basal and follow-up participants' demographic, anthropometrics, and clinical characteristics with and without MetS/T2D are showed in the Table S1. Prevalence of REE reduction (categorical variable) after 1 year was not significantly different between groups (48 vs. 46%, *p* = 0.56).

Table 1 shows the basal characteristics of the whole population according to RQ categories.

**Table 1:** Baseline participant's demographic, anthropometric, and clinical characteristics of the population according to low and high respiratory quotient.

**Variables**	**Low RQ (≤ 0.91) (*n* = 60)**	**High RQ (>0.91) (*n* = 28)**	***p-value***
Age (years)	60 (12)	61 (12)	0.69
BMI (Kg/m^2^)	28.9 (6)	27.3 (4)	0.11
WC (cm)	95 (11)	89 (9)	0.022
HC (cm)	104 (13)	103 (8)	0.68
SBP (mmHg)	126 (15)	123 (17)	0.61
DBP (mmHg)	77 (10)	75 (8)	0.23
RQ	0.82 (0.05)	0.97 (0.04)	< 0.001
REE (kcal)	1321 (229)	1180 (170)	0.002
Glucose (mg/dl)	93 (8)	91 (12)	0.46
Total Cholesterol (mg/dl)	209 (44)	214 (42)	0.64
Triglycerides (mg/dl)	103 (49)	102 (49)	0.94
HDL Cholesterol (mg/dl)	70 (30)	69 (25)	0.84
LDL Cholesterol (mg/dl)	131 (35)	134 (39)	0.76
**PREVALENCE**			
Male (%)	28	17	0.30
Smokers (%)	21	28	0.59
Physical activity (%)	25	14	0.50
Hyperlipidemia (%)	45	52	0.65
Lipid lowering agents (%)	43	50	0.64
Hypertension (%)	67	45	0.10
Antihypertensive agents (%)	65	45	0.10
Pts with 1-yr 5% weight loss (%)	4	11	0.20
Pts with 1-yr REE reduction (%)	50	44	0.46

*BMI, body mass index; WC, waist circumference; HC, hip circumference; SBP, systolic blood pressure; DBP, diastolic blood pressure; REE, resting energy expenditure; RQ, respiratory quotient; HDL, high density lipoprotein; LDL, low density lipoprotein*.

Participants in the High RQ group (>0.91) had a lower WC (*p* = 0.022) and REE (*p* = 0.002), than those in the Low RQ group (≤ 0.91). All other factors were not significantly different between groups.

**Figure 2:**
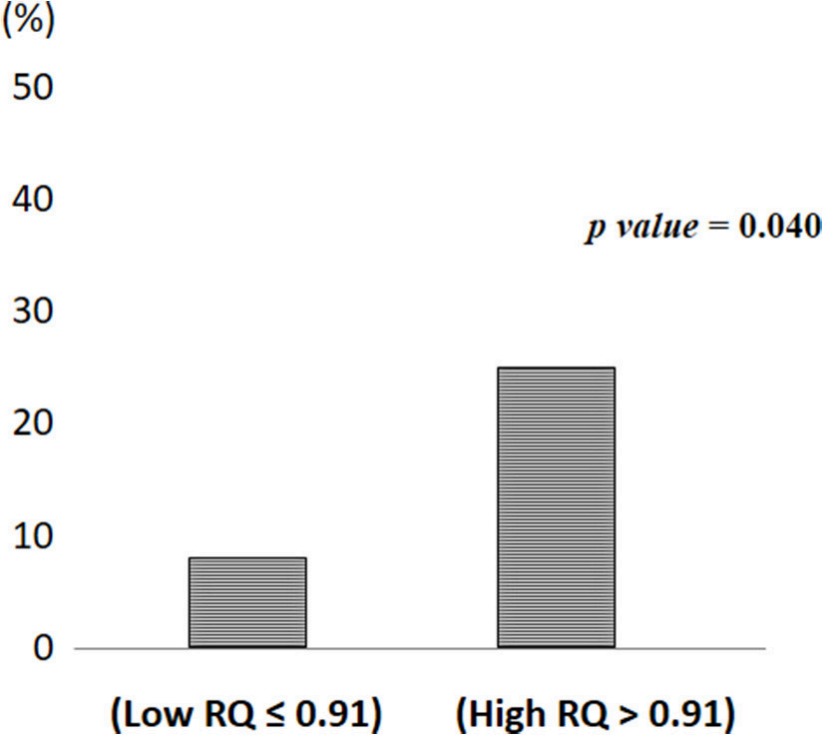
Metabolic syndrome/Type 2 diabetes incidence according to low and high respiratory quotient.

Figure 2 depicts the incidence of MetS/T2D according to High and Low RQ. Participants in High RQ group had a higher incidence (25%) than those in the Low RQ group (8%), (*p* = 0.04).

At univariate analysis, the factors which correlated with MetS/T2D were ΔW > 5% (categorical, *p* = 0.07, *r* = 0.19) and RQ categories (*p* = 0.034, *r* = 0.22) while no association was found with age and other basal parameters such as BMI, WC, weight, FM, RQ, REE, glucose, total cholesterol, HDL, Triglycerides, creatinine, SBP, and DBP (Table not shows). Furthermore, no association was found with WC increase (binary). In addition, RQ correlated only with REE (*p* < 0.001, *r* = −0.37) and not correlated with ΔW > 5% (*p* = 0.26, *r* = 0.12).

**Figure 3:**
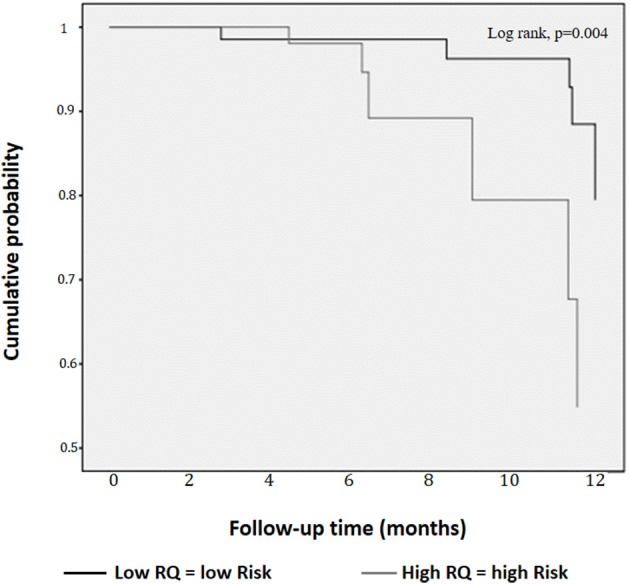
Kaplan-Meier curves for cumulative probability of metabolic syndrome/Type 2 diabetes according to low and high respiratory quotient.

As Figure 3 shows, the participants in the High RQ group had a greater probability of having MetS/T2D after 1 year than those in the Low RQ group (log Rank (Mantel-Cox) χ^2^-test = 8.44; df 1; *p* = 0.004).

The relationships between High RQ and MetS/T2D risk (*p* = 0.029; χ^2^ 14.09, Table 2), did not appear to be affected by gender, all drugs, REE and ΔW > 5% as estimated by Cox regression (p equal to 0.67; 0.25; 0.10; 0.50; 0.86, and 0.23 respectively, Table 2).

**Table 2:** Cox proportional hazards models for the risk of Metabolic Syndrome/Type 2 Diabetes.

**Variables**	**Multivariate analysis**	
	**HR (95% CI)**	***p-value***
Gender	1.443 (0.257–8.122)	0.67
Lipid lowering agents	0.446 (0.11–1.807	0.25
Antihypertensive agents	4.08 (0.739–22.518)	0.10
Oral hypoglycemic agents	2.119 (0.235–19.087)	0.50
REE	1 (0.996–1.005)	0.86
ΔW > 5%	3.9 (0.407–38.061)	0.23

*REE, resting energy expenditure; ΔW, weight increase >5%. Predictor, High RQ; p = 0.029; X^2^ = 14.09*.

When we performed the Kaplan-Meier curves to identify a different probability of having MetS/T2D after 1 year between participants who were or not Weight Stable (ΔW > 5%), no significantly difference was found (log Rank (Mantel-Cox) χ^2^-test = 3.33; df 1; *p* = 0.07).

## Discussion

The notion that whole body fat oxidation is impaired or insufficient to match the dietary fat load is central in the etiology of insulin resistance and T2D (13). We, thus, hypothesized that RQ measurement would predict the onset of MetS and T2D. In this study we found that individuals with a basal RQ >0.91 had a greater probability of having MetS/T2D after 1 year than those with RQ ≤ 0.91 (Figure 2). We did not find a different probability between subjects who were or not weight stable over a 1 year period. In this short period of time, a High RQ was the only predictor of MetS/T2D.

Our results are in line with a number of previous studies. It has been demonstrated that muscle obtained from patients with T2D has a reduced capacity for fat oxidation in culture (14).

In another study, ZDF rats displayed a significantly higher RQ than their lean littermates during hyperinsulinemia and, as the insulin levels declined, the RQ gradually decreased below the value of the lean animals (15).

Several data link MetS and diabetes to muscle mitochondrial dysfunction (16–18). When nutrient oxidation is inefficient, the ratio of ATP production/oxygen consumption is low, leading to an increased production of superoxide anions. These molecules stimulate proinflammatory processes which may contribute to metabolic and cardiovascular abnormalities.

However, studies in which fat oxidation, assessed by measuring RQ in humans, is associated with incident 1-year MetS or T2D are lacking., Our study is, thus, original and may have a relevant impact from a diagnostic and prognostic point of view.

Agents that reduce elevated FFAs, such as the thiazolidinediones (TZDs), have been shown to improve insulin sensitivity in muscle, liver, and adipose tissues (19, 20). Since it is also possible to manipulate fat oxidation, reduce RQ, and increase insulin sensitivity in humans (21), our study may also have a therapeutic impact.

FFAs represent a major link between obesity and diabetes (3). However, large-scale epidemiological studies have demonstrated a lack of association between plasma FFAs and measures of obesity (22–24). Other research have highlighted the heterogeneity of metabolic risk among obese individuals (25, 26), thus suggesting that weight (or fat mass) gain in adulthood may be only one of the mechanisms leading to an FFA increases and MetS risk (27).

Visceral fat is viewed as a key factor for causing insulin resistance (28), due to its greater catecholamine-stimulated lipolysis and direct drainage via the portal vein to the liver. However, visceral fat accounts for < 15% of total body fat and contributes < 15% of systemic FFAs (29). Furthermore, it has been demonstrated that some ethnic populations are more or less susceptible to visceral fat accumulation for a given amount of total body fat (30, 31).

Our finding are in line, to some extent, with these mechanism. With the univariate analysis, we demonstrate that MetS/T2D are not correlated with basal BMI, WC, or WC increase over 12 months and, despite its correlations with a weigh increase of >5%, the Kaplan-Meier curves showed no differences in incident MetS/T2D between participants who were or not weight stable over 1 year. This finding suggests a lack of association between incident Met/T2D and weight increase or other measures of obesity over 1 year. Furthermore, in our study, fat oxidation (RQ) and ΔW > 5% were not correlated (*p* = 0.26, *r* = 0.12, data not shown).

Despite the fact that abdominal obesity is a highly prevalent feature of the MetS, the causal factors implicated with MetS onset are not fully understood.

There is evidence that adipose tissue is not only specialized in the storage and mobilization of lipids but that it is also a notable endocrine organ releasing numerous cytokines, including proinflammatory molecules (32). Inflammation may therefore play a role in the onset of MetS and diabetes. However, a genetic predisposition in term of a low sympathetic activity and an impairment of the ACC/CPT I axis may well, together with environmental triggers, increase the susceptibility to these syndromes (5). Another causal factor may be the deficit in the capacity of subcutaneous fat to store excess energy resulting in increased accumulation of fat at undesired sites and MetS onset (33).

Regardless of the causative factors, it is clear that reduced fat oxidation is a convergence point for all these causal factors. RQ measurement could thus became a new tool for the prediction of MetS/T2D onset in the short term (6).

The novelty of our finding lies in the fact that there is no need to wait for anthropometric variations to predict the risk of MetS/T2D in an individual, as a single measurement of the RQ during fasting can suggest whether that subject is at risk.

Our results are also reinforced by the finding of a 11% prevalence of participants reporting at least a 5% weight reduction after 1 year in the High RQ group and 4% in the Low RQ group (Table 1), as well as a lower WC in the High than the Low RQ group. The combinations of component that included the central obesity component are more strongly associated with diabetes risk than others with the same number of metabolic abnormalities (34). Our results could thus be considered as a conservative estimate of the relationship between RQ and MetS/T2D risk. One may speculate that a lower REE in the High RQ than in Low RQ group would be implicated in MetS/T2D onset. However, we found the same prevalence of participants with some degree of REE reduction after 1 year in accordance with RQ categories (*p* = 0.46; Table 1). In addition, in the univariate analysis MetS/T2D and REE were not correlated and we found the same prevalence of participants with REE reduction in the group with and without MetS/T2D (48 vs. 46%, *p* = 0.56; Table S1).

These results confirm a previous cross-sectional study by our group in which RQ was significantly higher in individual with MetS and T2D than in those metabolically healthy overweight/obesity (8). In this previous study, the three groups had the same BMI but different RQ. Furthermore, as in other studies, in that population we demonstrated an association between RQ and HOMA-IR (8). One particular strength of the present work is that the study was performed in the same place, with the same conditions. Conversely, a limitation of this study is the small sample size, due to the difficulty in enrolling individuals without diabetes, MetS, dietary supplements or not taking any drug, with two complete consecutive nutritional examination. However, it has been reported that it is often better to test a new research hypothesis in a small number of subjects first (35). Of course, if an association is found, it just need to be interpreted carefully because it derives from a hypothesis-generating study and a larger confirmatory study is needed.

In this population, despite the use of lipid lowering agents, serum lipids were high. This is not a surprise. In subjects with low-intermediate cardiovascular risk, as in our population, despite evidence of benefit, the absolute risk reduction with lipid lowering therapy is expected to be low, and clinicians are often faced with the decision of whether or not to prescribe a lipid-lowering medication or to increase the dose. Furthermore, one of the problems associated with reaching the LDL-C target during statin treatment is the emergence of side effects. In relation to the prevalence of hypertension, previous studies showed that high blood pressure is by far the most prevalent single component of the metabolic syndrome and affects often more than 70% of all participants as single component (36, 37).

Our results are plausible because, in the group with basal RQ >0.91, the mean value was ~0.97. It has been reported that after an overnight fast, RQ is close to 0.85 (38, 39), thus, 0.96 is a high RQ value.

Currently, it is not known whether improving RQ will ultimately prevent the development of MetS or diabetes. However, it is notable that interventions that are known to be effective in preventing diabetes, including weight loss and treatment with insulin-sensitizing oral antidiabetic agents, also improve FFA and RQ in animal model (15). It has been demonstrated that ascorbic acid directly stimulates carnitine synthesis, β-oxidation of fatty acids, and reduces triglyceride accumulation (40) and that increased oxidative stress in accumulated fat is an important pathogenic mechanism of obesity-associated metabolic syndrome. Thus, in the future, clinicians will do better than recommending a conventional diet (low-fat or low-GI/GL diets) for prevention of type 2 diabetes but they will probably be able to modulate fat oxidation with specific nutrients and drugs.

In conclusion, impaired whole body fat oxidation plays a central role in the etiology of insulin resistance. Excessive FFA enhances glucose production by the liver and exacerbates the already existing hyperinsulinemia leading, through their lipotoxic effect, to cell failure, and overt T2D.

It is important for MetS and diabetes to be diagnosed early so that treatment can be started as soon as possible in order to prevent the metabolic and vascular complications. To our knowledge, this is the first time that a greater probability of having MetS/T2D after 1 year was found in individuals with a basal RQ >0.91 than those with RQ ≤ 0.91. Consequently, if confirmed by long-term studies, RQ assessed by indirect calorimetry could thus became a particularly useful tool in the diagnosis of MetS and diabetes.

## Author Contributions

TM and AP were responsible for study design, data analysis, and manuscript writing. YF and RP were responsible for enrollment. EM, YF, and SR were responsible for nutritional assessment. PD, RG, CG, and AC revised nutritional data and statistical analysis. All authors approved final manuscript.

### Conflict of Interest Statement

The authors declare that the research was conducted in the absence of any commercial or financial relationships that could be construed as a potential conflict of interest.
