# Supplementary material for: Lipid Oxidation Assessed by Indirect Calorimetry Predicts Metabolic Syndrome and Type 2 Diabetes
Source: Front Endocrinol (Lausanne). 2019 Jan 10;9:806. doi: 10.3389/fendo.2018.00806 (PMC6335247; doi:10.3389/fendo.2018.00806)
Supplement: Supplementary file 1 [file Table_1.DOCX]

| Supplemental Table 1*- Participant’s demographic, anthropometric and clinical characteristics of the population with or without Metabolic Syndrome/Type 2 Diabetes* | | | | | |  |
| --- | --- | --- | --- | --- | --- | --- |
| Variables | | Without MS/T2DM  (n=76) | | With MS/T2DM  (n=12) | *p-value* |  |
| ***Baseline characteristics*** | | | | | |  |
| Age (years) | | | 59 (12.6) | 65 (7) | 0.17 |  |
| BMI (Kg/m^2^) | | | 28.4 (5) | 27.8 (4) | 0.75 |  |
| WC (cm) | | | 92.5 (11) | 94.3 (10.5) | 0.64 |  |
| REE (kcal) | | | 1274 (216) | 1298 (257) | 0.73 |  |
| RQ | | | 0.87 (0.08) | 0.91 (0.09) | 0.16 |  |
| Glucose (mg/dl) | | | 91.7 (9) | 96.0 (10) | 0.15 |  |
| ***Follow- up characteristics*** | | | | | |  |
| Follow-up time (months) | | | 7.8 (3) | 8.7 (3) | 0.94 |  |
| BMI (Kg/m^2^) | | | 27.6 (5) | 27.7 (4) | 0.95 |  |
| WC (cm) | | | 92.5 (11) | 94.3 (10.5) | 0.22 |  |
| HC (cm) | | | 104 (10) | 113 (7) | 0.48 |  |
| SBP (mmHg) | | | 120 (12) | 132 (12) | 0.010 |  |
| DBP (mmHg) | | | 73 (9) | 77 (9) | 0.22 |  |
| Glucose (mg/dl) | | | 92 (11) | 100 (10) | 0.022 |  |
| Total Cholesterol (mg/dl) | | | 198 (38) | 187 (41) | 0.42 |  |
| Triglycerides (mg/dl) | | | 98 (54) | 125 (50) | 0.12 |  |
| HDL Cholesterol (mg/dl) | | | 64 (21) | 56 (36) | 0.46 |  |
| LDL Cholesterol (mg/dl) | | | 120 (35) | 120 (38) | 0.07 |  |
| ***Prevalences at Follow-up**** | | | | | | |
| Males (%) | 22 | | | 42 | 0.16 | |
| Smokers (%) | 27 | | | 33 | 0.73 | |
| Physical activity (%) | 26 | | | 50 | 0.30 | |
| Hyperlipidemia (%) | 47 | | | 33 | 0.53 | |
| Lipids lowering agents (%) | 42 | | | 33 | 0.50 | |
| Hypertension (%) | 29 | | | 75 | 0.007 | |
| Antihypertensive agents (%) | 26 | | | 73 | 0.006 | |
| Diabetes (n)  Oral hypoglycemic agents (n) | 0  0 | | | 2  2 | 0.017  0.017 | |
| Pts with 1-yr 5% weight loss (%) | 4 | | | 18 | 0.13 | |
| Pts with 1-yr REE reduction (%) | 48 | | | 46 | 0.56 | |
|  |  | | |  |  | |
| *Note.* MS/T2DM = Metabolic Syndrome and Type 2 Diabetes; BMI = body mass index; WC = waist circumference; HC = hip circumference; SBP = systolic blood pressure; DBP = diastolic blood pressure; REE = resting energy expenditure; RQ= respiratory quotient; HDL = high density lipoprotein; LDL = low density lipoprotein. | | | | | |  |
